# Potential of microbial-derived biosurfactants for oral applications–a systematic review

**DOI:** 10.1186/s12903-024-04479-0

**Published:** 2024-06-19

**Authors:** Z. Khairunnisa, N. Tuygunov, A. Cahyanto, W. H. Aznita, I. A. Purwasena, N. S.M. Noor, N. H. Azami, M. N. Zakaria

**Affiliations:** 1 Department of Restorative Dentistry, Faculty of Dentistry, Universiti Malaya, Kuala Lumpur, 50603 Malaysia; 2 Department of Oral Biology, Faculty of Dentistry, University of Jenderal Achmad Yani, Cimahi, 40525 Indonesia; 3 Department of Oral & Craniofacial Sciences, Faculty of Dentistry, Universiti Malaya, Kuala Lumpur, 50603 Malaysia; 4 Department of Microbiology, School of Life Sciences and Technology, Institut Teknologi Bandung, Bandung, 40132 Indonesia

**Keywords:** Antimicrobial, Biofilm, Minimum inhibitory concentration, Oral health, Oral pathology, Surface-active agent

## Abstract

**Background:**

Biosurfactants are amphiphilic compounds produced by various microorganisms. Current research evaluates diverse types of biosurfactants against a range of oral pathogens.

**Objectives:**

This systematic review aims to explore the potential of microbial-derived biosurfactants for oral applications.

**Methodology:**

A systematic literature search was performed utilizing PubMed-MEDLINE, Scopus, and Web of Science databases with designated keywords. The results were registered in the PROSPERO database and conducted following the PRISMA checklist. Criteria for eligibility, guided by the PICOS framework, were established for both inclusion and exclusion criteria. The QUIN tool was used to assess the bias risk for in vitro dentistry studies.

**Results:**

Among the initial 357 findings, ten studies were selected for further analysis. The outcomes of this systematic review reveal that both crude and purified forms of biosurfactants exhibit antimicrobial and antibiofilm properties against various oral pathogens. Noteworthy applications of biosurfactants in oral products include mouthwash, toothpaste, and implant coating.

**Conclusion:**

Biosurfactants have garnered considerable interest and demonstrated their potential for application in oral health. This is attributed to their surface-active properties, antiadhesive activity, biodegradability, and antimicrobial effectiveness against a variety of oral microorganisms, including bacteria and fungi.

## Introduction

Biosurfactants have recently attracted attention in biomedical research [1]. The demand for innovative solutions that prioritize eco-friendly and biobased polymeric surfactants is steadily rising. This growing concern is driven by the need for biodegradability and sustainability, which has prompted the development of technologies utilizing microbial sources [2, 3]. Biosurfactants or microbial-derived surfactants are surfactants that are produced by various microorganisms [4]. It has amphiphilic properties characterized by a hydrophilic head region (either polar or non-polar) and a hydrophobic tail region (such as lipid or fatty acid) [5, 6]. Biosurfactants have numerous advantages over chemical surfactants, including being less toxic, having a higher biodegradability, being environmentally friendly, having a higher foaming capability, being highly selective, and having specific activity at extreme pH, temperature, and salinity [7, 8]. They also bear the designations “eco-friendly”, “sustainable”, “bio-based”, or “green” materials [9].

Biosurfactants are classified into two classes based on their molecular weight: low molecular weight (LMW) and high molecular weight (HMW) [10]. Glycolipids and lipopeptides are examples of low molecular weight biosurfactants, such as rhamnolipids and surfactin, while phospholipids, lipoprotein, and emulsan are examples of high molecular weight biosurfactants [11, 12]. However, the market for commercially available biosurfactants is quite limited, with only a few options, such as surfactin, sophorolipids, and rhamnolipids [7].

Biosurfactants have been recognized as having a wide range of potential applications in various industries, including agriculture, food, cosmetics, pharmaceuticals, and petroleum [13–15]. Numerous studies have been conducted on biosurfactants and their prospective applications in environmental and biomedical fields as antimicrobials, antiadhesive/antibiofilm agents, antivirals, immune modulators, anticancer, wound-healing promoting agents, and drug delivery agents [16–18]. Biosurfactants also have the potential to be used in oral and dental infections [13]. The essential role of biosurfactant properties, including their ability to inhibit microorganisms and modify surface energy, has been well-established in controlling the formation and proliferation of biofilm [19–21].

The oral cavity comprises a diverse array of bacteria and fungi, commonly referred to as oral flora, which contribute to forming a complex oral ecosystem [22, 23]. They also contribute to the formation of an oral biofilm. Biofilm infections cause the majority of oral and dental pathogenic infections [24]. Biofilms are organized aggregates of microorganisms living in an extracellular polymeric matrix microbially produced and irreversibly attached to non-living or living surfaces [25]. Biofilm formation occurs in several common steps: initial contact/attachment to a surface, followed by micro colonization, maturation and formation of biofilm structures, and finally, biofilm detachment/dispersion [14, 15]. One of the most investigated biosurfactants is rhamnolipids. Abdollahi et al. reported that Rhamnolipids can reduce the adhesion of *Streptococcus mutans* on polystyrene surfaces and disrupt its preformed biofilm [26].

Similarly, Elshikh et al. also found that rhamnolipids from non-pathogenic *Burkholderia thailandensis* E264 revealed potent abilities to eradicate mature biofilm of some oral pathogens (*Streptococcus oralis*, *Actinomyces naeslundii*, *Neisseria mucosa*, and *Streptococcus sanguinis*) [27]. The complexity and diversity of this mature biofilm consist of numerous microenvironments [28] and can resistant to antimicrobial agents than planktonic cells [29]. This investigation has indicated that the utilization of biosurfactants for oral health applications is still in its initial phases. However, the available literature in this domain holds promise and is continually advancing. Hence, this systematic review aims to explore the potential of microbial-derived biosurfactants for oral applications.

## Methods

### Search strategy

**Fig. 1:**
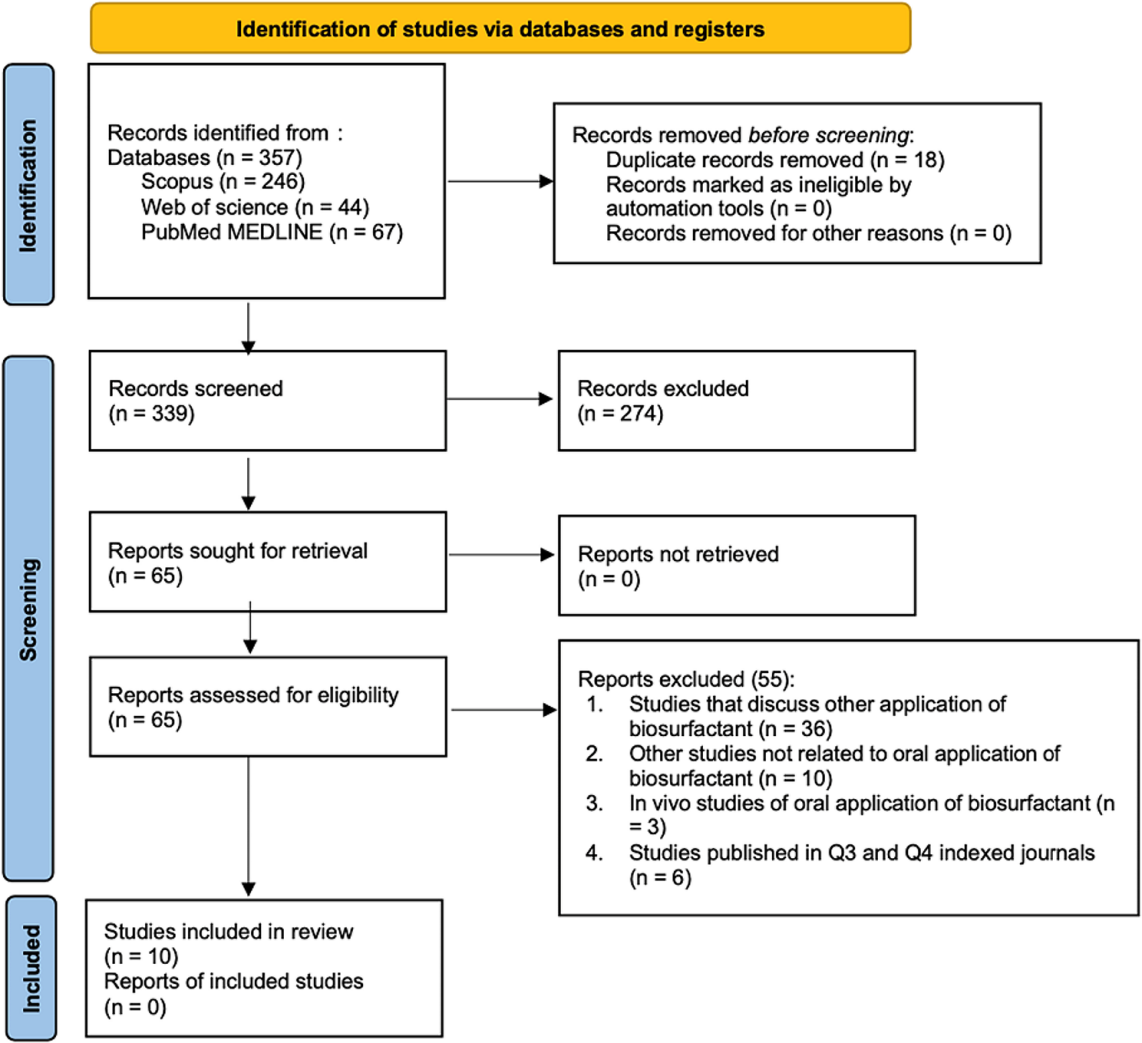
The outline of the article screening procedure in the PRISMA flowchart

This systematic review followed the guidelines outlined in the Preferred Reporting Items for Systematic Reviews and Meta-analysis (PRISMA) statement [30]. Two independent reviewers (K.Z and T.N) conducted a comprehensive search in three electronic databases: Scopus, Web of Science, and PubMed MEDLINE, utilizing the keywords described in Table 1. The titles and abstracts of the studies identified during the search were independently reviewed by both researchers (K.Z and T.N), and any discrepancies were resolved through discussion. Subsequently, the studies that met the inclusion and exclusion criteria were thoroughly examined. The search process included specific limitations on language, study design, and publication year. The complete search strategy employed in the Scopus, Web of Science, and PubMed MEDLINE databases can be found in Fig. 1.

**Table 1:** Keywords used in searching for the appropriate article

Keywords	
(“biosurfactant” OR “microbial surfactant”) AND (“oral pathogen” OR “oral bacteria” OR “oral disease” OR “oral application”) AND (“in vitro” OR “experimental study” OR “quasi-experimental study”)	

### Study selection

A total of 357 articles were retrieved from the search conducted in three electronic databases using the specified keywords. Two independent reviewers (K.Z and T.N) conducted the selection process, reviewing the complete list of articles and identifying potentially relevant papers based on title and abstract screening. Subsequently, the full texts of these selected articles were thoroughly examined to determine their eligibility based on the inclusion and exclusion criteria. Only articles published in English within the past 10 years and in journals categorized as Q1 and Q2 were included in the analysis as shown in Table 2. Papers in Q3 and Q4 are omitted due to the suboptimal clarity and quality of the images presented in the journal. This could potentially challenge the process of analysis. In disagreements, the reviewers engaged in discussions until a consensus was reached.

### Eligibility criteria

This systematic review has been registered with the National Institute for Health Research PROSPERO, International Prospective Register of Systematic Reviews, under the registration number CRD42023426727. The eligibility criteria for each type of study were determined based on specific characteristics, including the use of PICOS (Problem/Population, Intervention, Comparison, Objective, Study design), as outlined in Table 2.

The risk of bias will be evaluated using the Quality Assessment Tool for In Vitro Studies (QUIN tool) to assess the quality of the included studies. The QUIN tool is a standardized approach that enables researchers to assess the risk of bias in individual in vitro studies, ensuring consistency in evaluating the risk of bias in in vitro studies included in systematic reviews and meta-analyses. This tool has been evaluated for content validity and consists of 12 criteria. Each of these criteria was assigned a score as follows: adequately specified = 2 points, inadequately specified = 1 point, not specified = 0 points, and not applicable (N/A) = criteria excluded from the calculation. The scores for these 12 criteria were then summed to derive a total score for a specific in vitro study. These cumulative scores were subsequently employed to categorize the in vitro study into one of three risk levels: high (< 50%), medium (50–70%), or low risk (> 70%). This categorization was determined using the formula: Final score = (Total score x 100) / (2 x number of criteria applicable) [31].

**Table 2:** The criteria for eligibility based on the PICOS framework

Component of PICOS question	Inclusion	Exclusion
Problem/Population	Studies that discuss the oral application of biosurfactant	Studies that discuss other applications of biosurfactant
Intervention	Studies related to the oral application of biosurfactant	Studies not related to the oral application of biosurfactant
Comparison	Oral application of biosurfactant	Other applications of biosurfactant
Outcome	In vitro studies of oral application of biosurfactant	In vivo studies of oral application of biosurfactant
Study Design	Experimental and quasi-experimental studies, articles published not more than ten years, papers in Q1 and Q2 journals	Reviews, systematic reviews, book or book chapters, and conferences, articles not in English, articles published more than ten years, papers in Q3 and Q4 journals

### Data extraction

The primary and secondary reviewers have reached a consensus to extract the necessary data from reputable scientific databases, including Scopus, Web of Science, and PubMed MEDLINE. Prior to data extraction, the keywords to be used for data search were clarified and approved by supervisors in advance. Reviewer 1 gathers data in .csv format and imports it into an Excel file to create a table. The table will contain seven columns: authors, title, publication year, source title, abstract, link (or DOI), and comments. Reviewer 2 independently performs a comparable task in a parallel process, following the same inclusion/exclusion criteria. This approach ensures the precise selection of papers and minimizes the risk of errors.

## Results

### Qualitative study

The searches conducted in Scopus, Web of Science, and PubMed MEDLINE using the specified keywords yielded 246, 44, and 67 results, respectively. In total, 357 articles were collected and organized using a reference manager (EndNote). After manually removing 18 duplicate articles, 339 selected articles remained. Out of these, 274 articles were excluded as they focused on different compounds or chemical surfactants, leaving 65 articles for further consideration. From the remaining 65 articles, 55 were subsequently excluded, resulting in a final selection of 10 articles that met the inclusion criteria, as shown in Table 3. These selected articles were published between 2016 and 2020 [27, 32–40]. Detailed reasons for exclusion can be found in Fig. 1.

**Table 3:** Articles Selected for Inclusion in this Systematic Review

Authors	Title	Year	Source Title / Index	DOI
Elshikh M. et al. [27]	Rhamnolipids From Non-Pathogenic *Burkholderia thailandensis* E264: Physicochemical Characterization, Antimicrobial and Antibiofilm Efficacy Against Oral Hygiene Related Pathogens	2017	New Biotechnology / Q1	10.1016/j.nbt.2016.12.009
Bouassida, Mouna et al. [32]	Potential Application of *Bacillus subtilis* SPB1 Lipopeptides in Toothpaste Formulation	2017	Journal Of Advanced Research / Q1	10.1016/j.jare.2017.04.002
Bucci A.R. et al. [33]	The Antimicrobial and Antiadhesion Activities of Micellar Solutions of Surfactin, CTAB, and Cpcl With Terpinen-4-Ol: Applications to Control Oral Pathogens	2018	World Journal of Microbiology and Biotechnology / Q2	10.1007/s11274-018-2472-1
Ciandrini E. et al. [34]	Characterization of Biosurfactants Produced by *Lactobacillus Spp.* and Their Activity Against Oral Streptococci Biofilm	2016	Applied Microbiology and Biotechnology / Q1	10.1007/s00253-016-7531-7
Elshikh M. et al. [35]	Rhamnolipids and Lactonic Sophorolipids: Natural Antimicrobial Surfactants for Oral Hygiene	2017	Journal of Applied Microbiology / Q2	10.1111/jam.13550
Farias J.M. et al. [36]	Mouthwash Containing A Biosurfactant and Chitosan: An Eco-Sustainable Option for The Control of Cariogenic Microorganisms	2019	International Journal of Biological Macromolecules / Q1	10.1016/j.ijbiomac.2019.02.090
Janek T. et al. [37]	In Vitro Efficacy of The Lipopeptide Biosurfactant Surfactin-C15 and Its Complexes with Divalent Counterions to Inhibit *Candida albicans* Biofilm and Hyphal Formation	2020	Biofouling / Q2	10.1080/08927014.2020.1752370
Tambone E. et al. [38]	Counter-*Acting Candida albicans*-*Staphylococcus aureus* Mixed Biofilm on Titanium Implants Using Microbial Biosurfactants	2021	Polymers / Q1	10.3390/polym13152420
Tambone E. et al. [39]	Rhamnolipid Coating Reduces Microbial Biofilm Formation on Titanium Implants: An in Vitro Study	2021	BMC Oral Health / Q1	10.1186/s12903-021-01412-7
Yamasaki R. et al. [40]	Rhamnolipids and Surfactin Inhibit the Growth or Formation of Oral Bacterial Biofilm	2020	BMC Microbiology / Q2	10.1186/s12866-020-02034-9

### Study characteristics

The articles in this systematic review focus on in vitro studies investigating the efficacy of various biosurfactants. The biosurfactants analyzed in this review are predominantly rhamnolipids [27, 35, 38, 39], followed by surfactin [33, 37, 40], lipopeptide [32], and sophorolipid [35], which are comparatively less explored in the selected studies. Other studies in this systematic review did not specify the specific type of biosurfactant utilized in their research [34, 36], as shown in Table 4.

**Table 4:** Different types of biosurfactants in this systematic review

Authors	Source of biosurfactant	Medium	Type of biosurfactant	Purification
Elshikh M. et al. [27]	*Burkholderia thailandensis* E264 (ATCC 700,388)	Nutrient Broth	Rhamnolipid	Yes
Bouassida, Mouna. et al. [32]	*Bacillus subtilis* SPB1 (HQ392822)	Luria-Bertani with glucose, yeast extract, ammonium sulfate, and other salts (KH_2_PO_4_, K_2_HPO_4_, MgSO_4_)	Lipopeptide	No
Bucci A.R. et al. [33]	-	-	Surfactin (Lipofabrik, Lille, France)	Yes
Ciandrini E. et al. [34]	• *Lactobacillus reuteri* DSM 17,938 (Reuflor, Italchimici, Italy)	Man Rogosa and Shape (MRS) broth	-	Yes
	• *Lactobacillus acidophilus* DDS-1 (Nutratec, Urbino, Italy)			
	• *Lactobacillus paracasei* B21060 (Floratec, Bracco, Italy)			
	• *Lactobacillus rhamnosus* ATCC 53,103			
	• *Lactobacillus rhamnosus* ATCC 7469*			
	• *Lactobacillus casei* ATCC 15,008*			
	• *Lactobacillus salivarius* ATCC 11,741*			
Elshikh M. et al. [35]	*Starmerella bombicola* lactone esterase overexpression strain	Corn Steep Liquor (CSL) (100 g l^-1^ glucose.H_2_O, 5 g l^-1^ dried CSL, 1 g l^-1^ K_2_HPO_4_, 4 g l^-1^ (NH_4_)_2_SO_4_, 05 g l^-1^, MgSO_4_.7H_2_O)	Lactonic sophorolipids (LSLs)	Yes
	-	-	JBR425 Rhamnolipid (MR) (Jeneil Biotech Inc., Saukville, WI)	Yes
Farias J.M. et al. [36]	*Pseudomonas aeruginosa* UCP0992	Distilled water containing 4% dregs from a vegetable oil refinery and 0.5% corn steep liquor	-	-
	*Bacillus cereus* UCP1615	Mineral medium: 0.1% KH_2_PO_4_, 0.1% KH_2_PO_4_, 0.02% MgSO_4_·7H_2_O, 0.02% CaCl_2_·H_2_O and 0.005% FeCl_3_·6H_2_O, 2% waste soybean frying oil and 0.12% KNO_3_		
	*Candida bombicola* URM3718	Distilled water containing 5% sugarcane molasses, 5% waste frying oil, and 3% corn steep liquor		
Janek T. et al. [37]	*Bacillus subtilis* #309	10 g l^-1^ of sucrose (POCH, Gliwice, Poland), 10 g l^-1^ of NaCl (POCH), 2 g l^-1^ of NH_4_NO_3_ (Chempur, Poland), 5 g l^-1^ of Na_2_HPO_4_ (POCH), 2 g l^-1^ of KH_2_PO_4_ (POCH), and 0.2 g l^-1^ of MgSO_4_ .7H_2_O	Surfactin	Yes
Tambone E. et al. [38]	*Pseudomonas aeruginosa* 89	Nutrient Broth II (Sifin Diagnostics GmbH, Berlin, Germany) and Siegmund–Wagner medium	Rhamnolipids	-
Tambone E. et al. [39]	*Pseudomonas aeruginosa* 89	Nutrient Broth II (Sifin Diagnostics GmbH, Berlin, Germany) and Siegmund–Wagner medium	Rhamnolipids	-
Yamasaki R. et al. [40]	-	-	Rhamnolipids (AGAE Technologies, LLC, OR, USA)	-
			Sodium surfactin (Kaneka, Osaka, Japan)	
* To be excluded from further investigation - The paper did not mention this information.				

### Outcome measures

In this systematic review, all included studies employ diverse methodologies to examine the effects of various biosurfactants on different oral microorganisms. These evaluations encompass an array of characteristics associated with each biosurfactant, including surface tension, Critical Micelle Concentration (CMC), and physicochemical characterization. Additionally, antimicrobial activity, antibiofilm activity, antioxidant activity, and other factors are assessed. Some studies also utilize imaging techniques to provide visual clarity to their findings. The outcomes of these studies suggest that the identified biosurfactants hold promise as potential ingredients in various oral-related applications, such as toothpaste [32] and mouthwash [36]. The comprehensive overview of the methods employed and outcomes obtained in the studies included in this systematic review is presented in Table 5.

**Table 5:** Overview of the methods employed and outcomes of studies included in this systematic review

Authors, Year	Type of biosurfactant	Microorganism tested	Test subtances	Methodology	Results
Elshikh M. et al. [27] (2017)	Rhamnolipid	• *Streptococcus oralis* (DSM-20,627) • *Actinomyces naeslundii* (DSM-43,013) • *Neisseria mucosa* (DSM-4631); • *Streptococcus sanguinis* (NCTC 7863)	• Mono-rhamnolipid (MonoRh) fraction • Di-rhamnolipid fraction (DiRh) • SLS (Sodium Lauryl Sulpahte) • Rhamnolipid mix with SLS (Sodium Lauryl Sulpahte) in 1:10 ratio	1. Production, extraction, and purification of rhamnolipids 2. Chemical characterization 3. Surface tension and Critical Micelle Concentration (CMC) 4. Antimicrobial activity 5. Antibiofilm studies 6. Bisbenzimide accumulation assay 7. Leakage of nucleic acid material 8. Detection of Reactive Oxygen Species (ROS) production	1. The yield of rhamnolipid extracted was 2.64 ± 0.14 g/L. Purification was done using UPLC–MS. 2. The chemical analysis indicates that *Burkholderia thailandensis* E264 produces a mixture of rhamnolipids consisting of a 3:1 ratio of di-rhamnolipids to monorhamnolipids. 3. Surface tension was 30 mN/m, and CMC was 125 mg/L. 4. The MIC of monorhamnolipid fractions against three different oral microorganisms (excluding *A. naeslundii*) ranged from 1.25 to 2.50 mg/ml, di-rhamnolipid fractions (excluding *A. naeslundii*) ranged from 0.15 to 1.25 mg/ml, and SLS combined with rhamnolipid ranged from 0.001 to 0.002 mg/ml. 5. The biofilms of *S. sanguinis*, *S. oralis*, *N. mucosa*, and *A. naeslundii* were significantly inhibited at rhamnolipid concentrations of 0.39 mg/m1(90%); 0.78 mg/ml (70%); 6.25 mg/ml (70%); and 12.5 mg/ml (50%) respectively. A surface pre-coated with rhamnolipid at a concentration of 6.25 mg/ml caused an inhibition of biofilm formation of 57% for *S. oralis*, 70% for *N. mucosa* and *A. naeslundii*, and 83% for *S. sanguinis*. A low rhamnolipid concentration of 0.19 mg/ml produced a significant inhibition of around 65% for *S. sanguinis*, 0.03 mg/ml of around 80% for *S. oral* and *A. naeslundii*, and 50% for *N. mucosa*. 6. For all the organisms investigated, a direct proportionality exists between the increase in rhamnolipid concentration experienced by the cells and the intracellular accumulation of bisbenzimide. 7. There was an increasing leakage of UV-absorbing material from the cells with increasing rhamnolipid concentration to all the microorganisms. 8. A dose-response for the ROS was detected in all the bacteria treated with rhamnolipid.
Bouassida, Mouna. et al. [32] (2017)	Lipopeptide	• *Escherichia coli* (ATCC 25,922) • *Enterococcus faecalis (*ATCC 29,212) • *Enterobacter sp Listeria monocytogenes* (ATCC 43,251) • *Klebsiella pneumoniae* (ATCC 13,883) • *Salmonella enterica* (ATCC 43,972) • *Salmonella typhinirium* (ATCC 19,430) • *Micrococcus luteus* (ATCC 4698)	Sodium Dodecyl Sulfate (SDS): SDS-based toothpaste SS: toothpaste without emulsifier Commercial toothpaste	1. Biosurfactant production 2. Formulation of toothpaste 3. Physico-chemical evaluation 4. Cleaning ability test 5. Antibacterial assay 6. Stability studies	1. The supernatant-free cells from B. subtilis SPB1 were precipitated and served as crude lipopeptides. 2. -Formula BIO-1: emulsifier with biosurfactant 0.5 g; sodium alginate 1 g; calcium carbonate 4 g; sodium chloride 1.5 g; sodium fluoride 0.5 g; glycerin 4 g -Formula BIO-2: emulsifier with biosurfactant 0.5 g; calcium carbonate 1.5 g; sodium chloride 1.5 g; sodium fluoride 0.5 g; glycerin 4 g 3. The desiccation loss of the biosurfactant-formulated toothpaste was between 22 and 30%. Biosurfactant-based toothpaste presented lower foaming ability (33%). The spreading ability test was 20 mm for BIO-1, indicating a low value equal to 16.5 mm. The water activity of BIO-1 and BIO-2 ranged from 0.22 to 0.28. 4. The formula BIO-1 and the commercial toothpaste had the same ability to clean stains. Formula 2 (BIO-2) showed a change in the color of eggs from yellow to brown. Due to the heterogeneity of formula 1, its high pH value, low spreading ability, and low cleaning efficiency, formula 1 was used for further experiments. 5. BIO-1 was very effective against the tested microorganisms except *E. coli*. The inhibition diameter was observed against *Enterobacter sp* (22 mm) and *Salmonella typhinirium* (20 mm), and *Listeria monocytogenes* (12.67 mm). BIO-1 was more effective than commercial toothpaste and SDS in inhibiting *Listeria monocytogenes*, *Klebsiella pneumoniae*, and *Salmonella typhinirium.* 6. The spreading power of all formulas did not change during storage. The foaming ability of BIO-1 was not stable. There was a decrease in the pH value of all formulas, except the biosurfactant-based toothpaste, and an increase in the water activity value of all formulated toothpaste.
Bucci A.R. et al. [33] (2018)	Surfactin	*Streptococcus mutans* (ATCC 25,175), *Streptococcus mitis* (ATCC 49,456), *Streptococcus salivarius* (ATCC 13,419), *Porphyromonas gingivalis* (ATCC 33,277), *Pseudomonas aeruginosa* (ATCC 27,853), *Staphylococcus aureus* (ATCC 25,923), *Escherichia coli* (ATCC 25,922), *Candida albicans* (ATCC 10,231)	Terpinen-4-ol (TP) Cetylpyridinium chloride (CPC) Cetyl trimethyl ammonium bromide (CTAB) (all of which with purity above 95% (from Sigma-Aldrich, Brazil))	1. Disk diffusion assay 2. Minimum inhibitory concentration 3. Determination of combination index (CI) for micellar solutions 4. Adhesion on test tubes	1. Inhibition was observed when surfactin was mixed with TP against *S. aureus, P. gingivalis*, and *E. coli*, while no inhibition was detected for surfactin against all microorganisms. 2. MIC of surfactin ranged from 22 µg/mL to 700 µg/mL, with no inhibition for *P.gingivalis*. 3. Combination index for surfactin and TP were synergism for *S. mutans* and *P.gingivalis*, slight synergism for *C. albicans* and *E. coli*, additive for *S. aureus*, and antagonism for *P. aeruginosa*, *S. salivarius*, and *S. mitis*. 4. The micellar solution of surfactin and TP showed very high enhancement in antiadhesion activity for two oral pathogens (*C. albicans* and *P. gingivalis*).
Ciandrini E. et al. [34] (2016)	Biosurfactant from *Lactobacillus*	*Streptococcus mutans* ATCC 25,175 and *Streptococcus oralis* ATCC 9811	1% SDS (common chemical surfactant)	1. Biosurfactants preparation and assessment of the antimicrobial activity by time-killing studies 2. Dialysis of biosurfactants 3. Characterization of dialyzed biosurfactant surface properties 4. Biofilm formation 5. Anti-biofilm effect of dialyzed biosurfactants	1. Biosurfactants from *L. reuteri* DSM 17,938, *L. acidophilus* DDS-1, *L. paracasei* B21060, and *L. rhamnosus* ATCC 53,103 showed the greatest antimicrobial activity against *S. mutans* and *S. oralis*. They were selected for further studies, and their excreted biosurfactants were purified through dialysis. 2. Biosurfactants excreted by *L. reuteri* DSM 17,938, *L. acidophilus* DDS-1, *L. rhamnosus* ATCC 53,103, and *L. paracasei* B21060 strains were dialyzed using 1a and 6 kDa methods. 3. A reduction of the interfacial tension from 47.92 to 34.81 mN/m was observed compared to MRS broth’s surface tension (53.0 mN/m). The highest emulsifying activity was obtained from the dialyzed 6 kDa biosurfactant of *L. paracasei* B21060 (61.11%). 4. All 1 and 6 kDa dialyzed BSFs effectively inhibited *S. mutans* ATCC 25,175 and *S. oralis* ATCC 9811 biofilm growth. Biomass analysis highlighted the dose-dependent effect of all the dialyzed biosurfactants (1 and 6 kDa), particularly remarkable in the case of biofilm formation inhibition of *S. oralis* ATCC 9811. 5. The 6 kDa dialyzed BSFs possess antibiofilm activity against *S. mutans* ATCC 25,175 and *S. oralis* ATCC 9811 with optical density of 0.329 (± 0.026) for BSF of *L. reuteri* DSM 17,938, 0.280 (± 0.009) for *L. acidophilus* DDS-1, 0.356 (± 0.025) for *L. rhamnosus* ATCC 53,103, and 0.261 (± 0.012) for *L. paracasei* B21060.
Elshikh M. et al. [35] (2017)	Lactonic sophorolipids (LSLs), Rhamnolipid	*S. mutans* (DSM- 20,523) *S. oralis* (DSM-20,627) *A. naeslundii* (DSM-43,013) *N. mucosa* (DSM-4631) *S. sanguinis* (NCTC 7863).	Mixed congener JBR425 Rhamnolipid (MR) monorhamnolipid and dirhamnolipid	1. Production, extraction, and purification of LSL 2. Rhamnolipids investigated in this study 3. Separation of rhamnolipid congeners 4. Determination of critical micelle concentration 5. Antimicrobial activity of biosurfactants 6. Combination effect of biosurfactant-antibiotic / antimicrobial on MIC 7. Biofilm studies 8. Co-incubation assay 9. Anti-adhesion assay 10. Biofilm disruption assay 11. Bisbenzimide accumulation assay	1. LSL analysis showed an overall content of lactonic sophorolipid > 99%, mainly consisting of 91.73% diacetyl C18:1 (MW 688.4 g mol^-1^), 5.75% diacetyl C18:2 (MW 686.3 g mol^-1^) and a smaller percentage (25%) of monodicetyl C18:1 (MW 646.4 g mol^-1^). 2. Relative abundance analysis of MR showed a 1 : 1 ratio of monorhamnolipid to dirhamnolipid congeners. 3. The purified monorhamnolipid fraction was comprised of over 99%, with the highest relative abundance of 68.87% for Rh-C10- C10 (MW 504.3 g mol^-1^). The dirhamnolipid purified fraction had content of > 98%, whereas Rh-Rh-C10-C10 (MW 650.3 g mol^-1^) had the highest relative abundance of 65.22% of the total sample. 4. CMCs values for Mono-RL, Di-RL, MR, and LSLs were 32, 31, 48, and 22 µg/ml, respectively. 5. The rhamnolipid mixture (MR) is more effective against all tested microorganisms than the purified monorhamnolipid and di-rhamnolipid fractions. LSLs have a better killing effect compared to MR. 6. A small biosurfactant concentration decreased the MIC for antibiotics and standard antimicrobials significantly compared to standard antimicrobial agents alone. 7. MR 0.2 mg/ml eliminated preformed biofilms of *S. oralis*, *S. sanguinis*, and *A. naeslundii*, while *N. mucosa* and *S. mutans* biofilms were inhibited by 0.4 and 1.0 mg/ml of MR. Biofilms of *S. oralis*, *S. sanguinis*, *N. mucosa*, and *A. naeslundii* were inhibited with 0.2 mg/ml LSLs, while they required 1.0 mg/ml to reduce the biofilm of *S. mutans*. 8. Using as little as 0.2 mg/ml, MR resulted in more than 80–90% growth inhibition for all microorganisms tested except for *S. mutans* (around 60%). Using 0.2 mg/ml of LSL caused 90% biofilm inhibition against all the organisms used. 9. Pre-coating experiment using 0.2 mg/ml LSL prevented more than 80% biofilm formation of all microorganisms investigated, except *S. mutans*, which required a higher concentration of 0.4 mg/ml to achieve 40% growth inhibition. 10. Biofilm disruption assessment demonstrated excellent potency of rhamnolipids and LSLs to restrict developing biosurfactants. 11. Increasing concentrations of rhamnolipids mixture (0.5–5– 50) mg/ml and LSL (0.25–2.5–25) mg/ml directly related to increasing dye concentrations in the bacterial cells.
Farias J.M. et al. [36] (2019)	Biosurfactant *Pseudomonas aeruginosa* (PB), *Bacillus cereus* (BB), *Candida bombicola* (CB)	*C. albicans* ATCC 1106 *S. aureus* ATCC 15,656 *E. coli* ATCC 25,922 *L. acidophilus* ATCC 4356 *S. salivarius* ATCC 25,975 *S. mutans* ATCC 25,175	Chitosan (extracted from the cell wall of *Mucor javanicus* (UCP 69)), Peppermint essential oil (POE)	1. Characterization of biosurfactants 2. Formulation of mouthwash 3. Determination of antimicrobial activity 4. Analysis of fraction inhibitory concentration 5. Evaluation of toxicity of formulated mouthwashes	1. The biosurfactants obtained from *C. bombicola*, *B. cereus*, and *P. aeruginosa* (respectively designated as CB, BB, and PB) were capable of reducing the surface tension of water from 70 mN/m to 30, 29, and 26.5 mN/m, respectively. 2. The formulation consists of biosurfactant, peppermint essential oil, and chitosan solution (diluted in 1% acetic acid), the solid components (sodium benzoate and sodium saccharine) in a fine powder, followed by distilled water to complete 100 mL. The pH of the formulation was adjusted to 7.0 with NaOH 1 N. 3. The biosurfactant PB was the most effective against *S. aureus*, *E. coli*, and *S. salivarius* (MIC: 20 µg/mL), while PB and CB had similar effects on *S. mutans* (MIC: 20 µg/mL). All biosurfactants exhibited uniform effects on *C. albicans* and *L. acidophilus* (MIC: 40 µg/mL). Combining biosurfactants with chitosan reduced MIC for all microorganisms. When combined with peppermint essential oils, the MIC for *C. albicans* either increased (CB + POE and BB + POE: 30 µg/mL) or remained the same (PB + POE: 20 µg/mL). The low MIC combination for *L. acidophilus* (20 µg/mL) was peppermint essential oil with PB. For *S. mutans*, only CB + POE maintained the biosurfactant’s MIC, with reductions observed in all other combinations. 4. The combinations of the CB and PB with chitosan demonstrated an additive effect on the majority of microorganisms tested and an indifferent effect on *E. coli* and *C. albicans*. The combinations of CB and BB with the peppermint essential oil exhibited an additive effect only on the gram-negative bacterium *E. coli* and an indifferent effect on the other microorganisms tested. 5. The results demonstrate that the test mouthwashes were classified as non-toxic to the fibroblast line, with cell inhibition rates lower than 20%. However, the mouthwash containing the biosurfactant extracted from *C. bombicola* + peppermint essential oil + chitosan exhibited moderate toxicity to the macrophage line (66% inhibition).
Janek T. et al. [37] (2020)	Surfactin (SF)	*Candida albicans* SC5314 *C. albicans* ATCC 10,231	Ca(II)-SF Mg(II)-SF Cu(II)-SF Zn(II)-SF	1. Purification and characterization of SF 2. Growth inhibition assays 3. In vitro anti-biofilm assay 4. Biofilm quantification 5. Hypha formation 6. Quantification of gene expression by quantitative real-time PCR (qRT-PCR) 7. Cellular surface hydrophobicity (CSH) assay	1. The crude mixture of biosurfactants was characterized by preparative reversed-phase high-performance liquid chromatography (RP-HPLC). 2. SF at concentrations from 0.075 to 1 mM (72 to 960 mg/ml) exhibited low growth inhibition (0–11%) of C. albicans SC5314. 3. SF and its metal(II) complexes inhibited biofilm formation in a dose-dependent manner when these compounds were added to C. albicans cells after a short initial adhesion period. SF at 1 mM (960 mg/ml) caused 85% inhibition. This effect was enhanced when metal (II)- SF complexes were used. 4. SF and its metal(II) complexes inhibited biofilm biomass production. The mature biofilm was estimated to be 38% when SF was added at the concentration of 1 mM (960 mg/ml). In addition, Ca(II)-SF, Mg(II)-SF, Cu(II)-SF and Zn(II)-SF (1 mM) reduced mature biofilms by 78%, 79%, 72% and 69%, respectively. 5. The cells treated with SF and metal(II)-SF complexes showed attenuated fluorescence density of HWP1- GFP, suggesting that the compounds had an impact on the expression of hypha-related genes. 6. The expression of the hypha-specific genes for planktonic and biofilm-forming cells were downregulated after exposure to SF and Mg(II)-SF. All metal(II)-SF complexes significantly altered the expression of hypha-specific and biofilm-related genes. 7. The relative CSH of untreated C. albicans cells was 0.67, and the CSH underwent a reduction in response to SF concentration. The best results were obtained for Mg(II)-SF, where the CSH was reduced to 0.23, 0.12, and 0.01 with exposure to 0.5, 0.75, and 1 mM of Mg(II)-SF, respectively.
Tambone E. et al. [38] (2021)	Rhamnolipids (R89BS)	*Candida albicans* ATCC ® 10,231 and *Staphylococcus aureus* ATCC ® 6538	-	1. Biosurfactant Production 2. Anti-Biofilm Activity of R89BS‐Coated Titanium Discs against Multi‐Species Biofilm 3. Eukaryotic Cell Viability Tests	1. The supernatant of crude biosurfactant was extracted three times with ethyl acetate, and the composition of the raw extract was confirmed by mass spectrometry analysis. 2. R89BS coating more effectively inhibited biofilm biomass than cell metabolic activity and viability. Quantitatively, R89BS-coated TDs demonstrated the highest ability to reduce biofilm formation at 24 h, with inhibitions of biofilm biomass, cell metabolic activity, and cell viability exceeding 90%. After 48 h, biofilm biomass and cell viability were inhibited by 36% and 29%, respectively, while metabolic activity showed a less pronounced effect with a 14% inhibition. 3. Cell viability of hOBs (Human primary osteoblasts) decreased below 70% at R89BS concentrations exceeding 50 µg/mL. Concentrations equal to or lower than 50 µg/mL showed no interference with hOBs growth, maintaining cell viability above 80%. No cytotoxic effects were observed on hOBs cultured in the eluate from R89BS-coated titanium.
Tambone E. et al. [39] (2021)	Rhamnolipids	*S. aureus* ATCC 6538 and *S. epidermidis* ATCC 35,984	Three commercially available titanium surfaces (M&P, L-L, and RBT)	1. Biosurfactant Production 2. Cytotoxicity of R89BS-coated TDs 3. Anti-biofilm activity of R89BS‐coating 4. Efficacy of R89BS-coating on commercial titanium surfaces	1. The supernatant of crude biosurfactant was extracted three times with ethyl acetate, and the composition of the raw extract was confirmed by mass spectrometry analysis. 2. The cytotoxicity assay showed no cytotoxic effect on human lung fibroblast cell lines when exposed to the TDs coated with 4 mg/mL R89BS eluate obtained from dynamic release conditions. 3. R89BS-coating was more effective in reducing *S. aureus* biofilm biomass. Conversely, it mainly inhibited *S. epidermidis* biofilm regarding cell metabolic activity. Quantitatively, R89BS-coated TDs exhibited the highest ability to reduce biofilm formation at 24 h for both *Staphylococcus* strains, with 98.6% and 94.3% inhibition of biofilm biomass and cell metabolic activity for *S. aureus* and 54.1% and 68.9% for *S. epidermidis*, respectively. 4. R89BS-coated samples inhibited *S. aureus* biofilm biomass by over 90%. For *S. epidermidis*, all coated surfaces showed significant biofilm inhibition compared to uncoated controls, with inhibition percentages ranging from 62 to 78%, depending on surface morphology.
Yamasaki R. et al. [40] (2020)	Rhamnolipids (AGAE Technologies, LLC, OR, USA) and Sodium surfactin (Kaneka, Osaka, Japan)	*A. actinomycetemcomitans* Y4 ATCC43718, *S. mutans* UA159 ATCC700610, *S. sanguinis* ATCC10556	-	1. Inhibitory effects on bacterial cell growth of rhamnolipids and surfactins 2. Inhibitory effects on biofilm formation of rhamnolipids and surfactins	1. Rhamnolipids significantly inhibited the growth of *S. mutans* UA159 and *S. sanguinis* ATCC10556; however, *A. actinomycetemcomitans* Y4 was unaffected. Alternatively, surfactin exhibited the highest inhibitory effect on *S. sanguinis* ATCC10556, whereas no effect was observed on *A. actinomycetemcomitans* Y4 and *S. mutans* UA159. 2. Rhamnolipid at 3.17 × 10 − 3 w/v% inhibited *A. actinomycetemcomitans* Y4 biofilm formation, with 93% inhibition at 0.013 w/v%. For *S. mutans* UA159, biofilm formation was inhibited by rhamnolipids at 6.35 × 10 − 3 w/v%, reaching near complete inhibition at 0.1 w/v%. In *S. sanguinis* ATCC10556, complete inhibition occurred at 6.35 × 10 − 3 w/v%, while biofilm formation increased 2-fold at 1.98 × 10 − 4 w/v%. Surfactin at 10.36 w/v% inhibited 90% of *A. actinomycetemcomitans* Y4 biofilm, but concentrations from 2.53 × 10 − 3 w/v% to 2.59 w/v% promoted biofilm formation up to 6-fold. *S. mutans* UA159 showed surfactin-induced biofilm promotion with no inhibitory effect. Surfactin concentrations > 2.53 × 10 − 3 w/v% caused near complete inhibition of *S. sanguinis* ATCC10556 biofilm formation.

### Bibliometric analyses

**Fig. 2:**
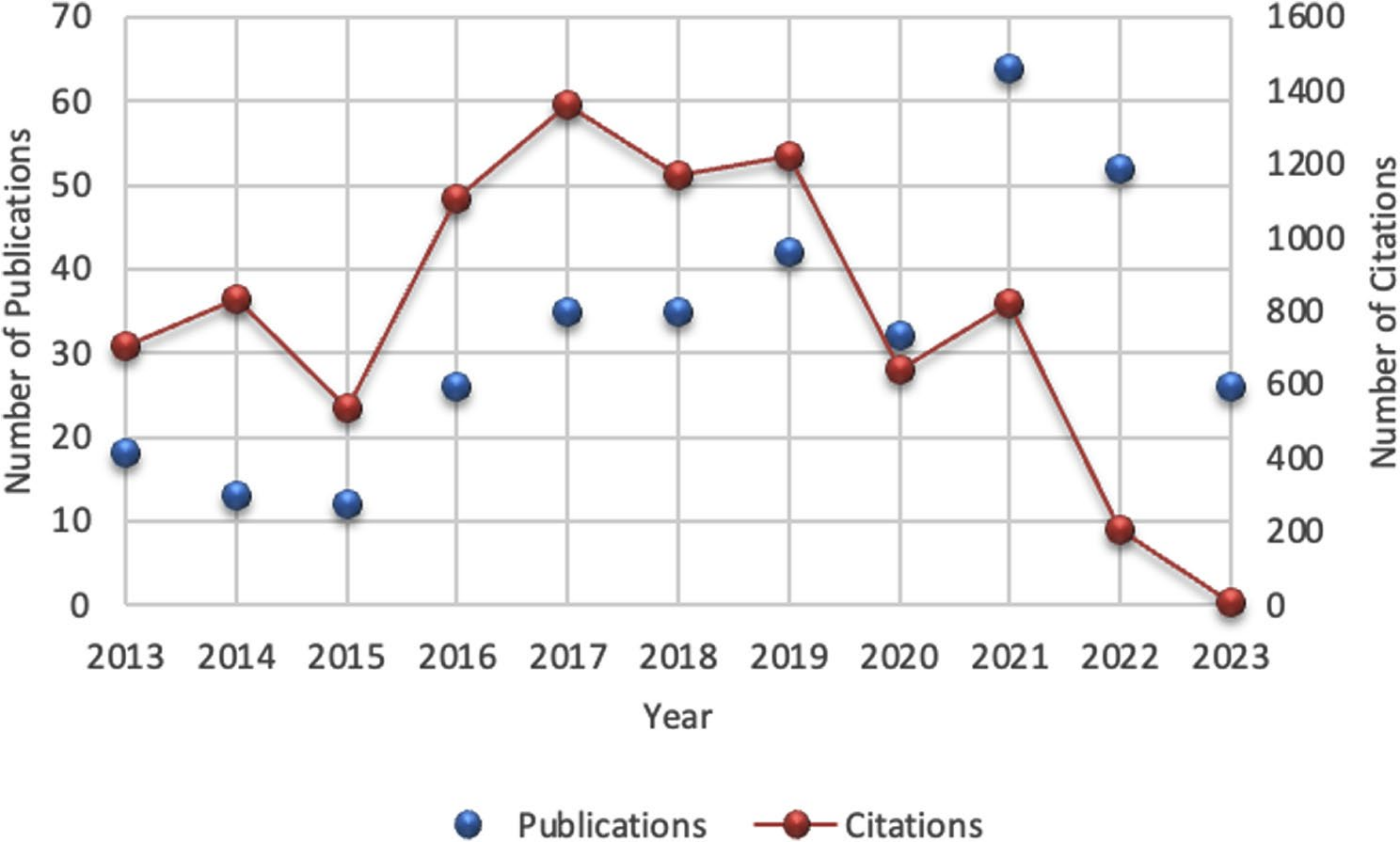
The total count of articles published and citations within ten years

A sum of 357 research articles and reviews were encompassed in the study. Figure 2 illustrates the distribution and the total of citations of these publications over ten years. The peak year for publications was 2021, with 64 articles published, while the year with the fewest publications was 2015, with only 12 articles published. Citation counts fluctuated between 707 citations in 2013 and only 6 in 2023 since the publication in 2023 is still in progress. The year with the most substantial citations was 2017, with a remarkable 1360 citations.

**Fig. 3:**
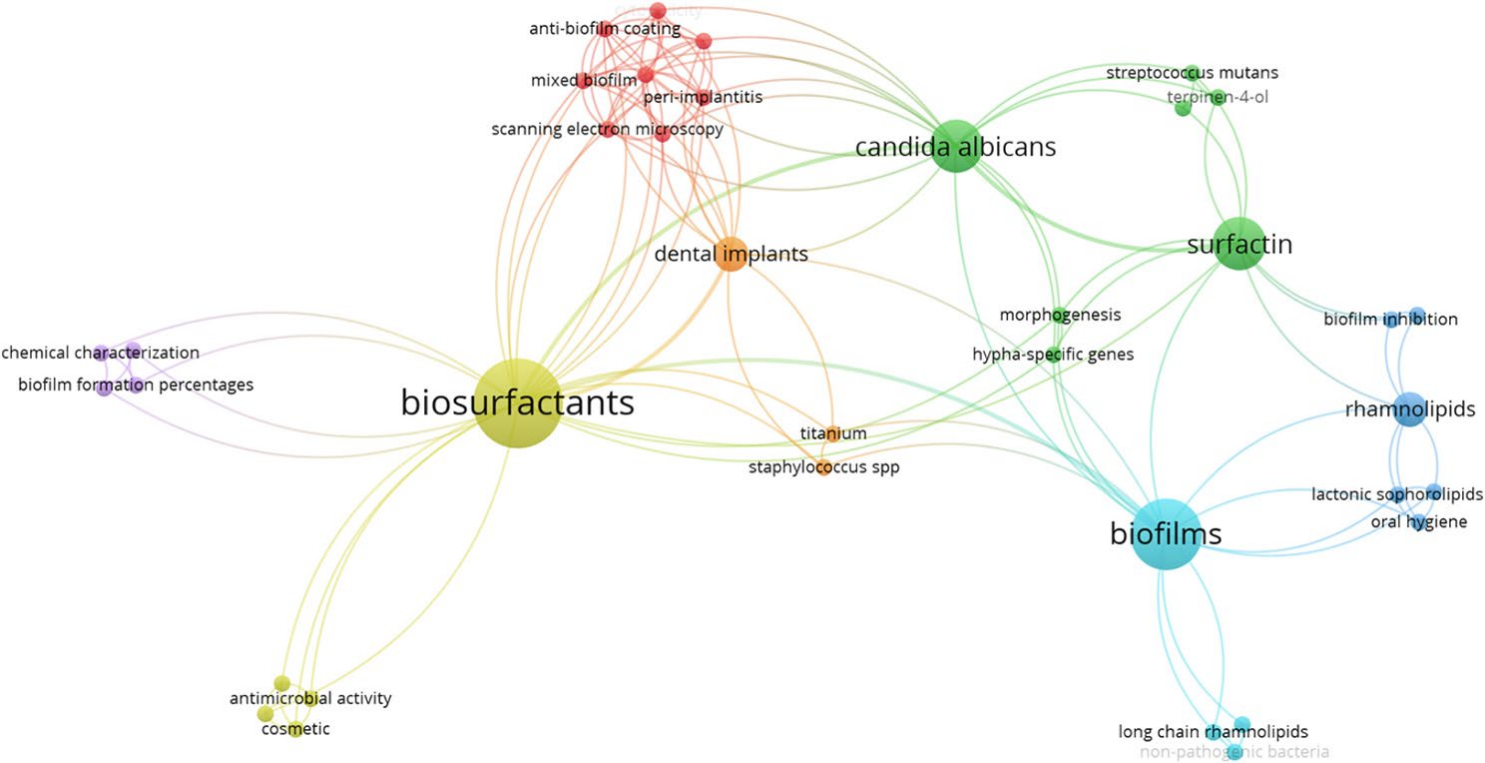
The co-occurrence of keywords from the ten selected articles. The proximity of two nodes in the graph indicates a higher number of co-occurrences between the corresponding keywords

A total of 37 keywords were extracted from the ten selected articles as seen in Fig. 3. The three most frequently used keywords were “biosurfactants,” “*Candida albicans*,” and “biofilms,” with respective total link strengths of 27, 19, and 16. Cluster analysis was performed on the co-occurrence of keywords from the ten selected articles, resulting in seven clusters. Cluster 1, comprising 8 keywords, included terms such as “anti-biofilm coating,” “cytotoxicity,” “fungal-bacterial biofilm,” “mixed biofilm,” and more. Cluster 2 mainly focused on terms related to “*Candida albicans*,” “hypha-specific genes,” “morphogenesis,” “*Streptococcus mutans*,” and others. Cluster 3 primarily concentrated on “biofilm inhibition,” “minimum inhibitory concentration,” “oral bacteria,” and other related terms. Cluster 4 consists of 4 terms such as “antimicrobial activity,” “biosurfactants,” and more. Clusters 5, 6, and 7 consist of 4, 4, and 3 terms respectively. Analyzing the keyword co-occurrences, it becomes evident that numerous in vitro experiments involving biosurfactants focused on evaluating their effectiveness against *Candida albicans* for inhibiting biofilm formation. Notably, surfactin emerged as the predominant type of biosurfactant utilized in these studies.

A total of 243 journals contributed to the collection of enrolled publications. Table 6 presents the top 10 journals that extensively covered the subject of “potential biosurfactant for oral application.” The Journal of Applied Microbiology emerged as the leading article regarding productivity, having produced the highest number of publications on this topic. Furthermore, it was also identified as the most influential journal, with the highest number of citations per paper to the subject matter.

**Table 6:** Top ten journals with the highest number of publications on oral application of biosurfactants between 2013 and 2023

Ranking	Journal	Publications, *N* (%)	Citations, *N* (%)	JIF (2023)
1	Journal of Applied Microbiology	10 (4.12)	472 (5.48)	4.72
2	Microbial Pathogenesis	8 (3.29)	415 (4.82)	3.98
3	Microorganisms	7 (2.88)	157 (1.82)	4.60
4	Frontiers in Microbiology	7 (2.88)	335 (3.89)	5.08
5	Applied Microbiology and Biotechnology	6 (2.47)	249 (2.89)	5.43
6	Antibiotics	5 (2.06)	53 (0.62)	4.93
7	Archives of Oral Biology	5 (2.06)	118 (1.37)	2.79
8	Biocatalysis and Agricultural Biotechnology	5 (2.06)	71 (0.82)	4.66
9	Biofouling	4 (1.65)	83 (0.96)	2.95
10	Molecules	4 (1.65)	32 (0.37)	4.71

### Risk of bias and quality assessment

Two independent reviewers (K.Z and T.N) assessed the risk of bias in this study using the Quality Assessment Tool For In Vitro Studies (QUIN) Tool [31]. The risk of bias in each study can be found in Table 7.

**Table 7:** Quality assessment of the included studies according to the QUIN tool for in vitro studies

No	Criteria	Elshikh M. et al. [27]	Bouassida, Mouna. et al. [32]	Bucci A.*R*. et al. [33]	Ciandrini E. et al. [34]	Elshikh M. et al. [35]	Farias J.M. et al. [36]	Janek T. et al. [37]	Tambone E. et al. [38]	Tambone E. et al. [39]	Yamasaki *R*. et al. [40]
1	Clearly stated aims/objectives	2	2	2	2	2	2	2	2	2	2
2	Detailed explanation of sample size calculation	1	2	1	2	1	2	2	2	2	1
3	Detailed explanation of sampling techniques	1	1	1	2	2	1	1	2	2	1
4	Details of comparison group	2	2	2	2	2	2	2	1	2	1
5	Detailed explanation of methodology	2	2	2	2	2	2	2	2	2	2
6	Operator details	0	0	0	0	0	0	0	0	0	0
7	Randomization	0	0	0	0	0	0	0	0	0	0
8	Method of measurement of outcome	2	2	2	2	2	2	2	2	2	2
9	Outcome assessor details	N/A	N/A	N/A	N/A	N/A	N/A	N/A	N/A	N/A	N/A
10	Blinding	N/A	N/A	N/A	N/A	N/A	N/A	N/A	N/A	N/A	N/A
11	Statistical analysis	2	2	0	2	2	1	1	2	2	0
12	Presentation of results	2	2	2	2	2	2	2	2	2	2
	Final score	70	75	60	80	75	70	70	75	80	55
	Category	Medium	Low	Medium	Low	Low	Medium	Medium	Low	Low	Medium

Five articles were classified as having a low risk of bias [32, 34, 35, 38, 39], while another five were categorized as having a medium risk [27, 33, 36, 37, 40]. Consequently, all the articles included in this review met or exceeded 50% of the assessed criteria.

## Discussion

**Fig. 4:**
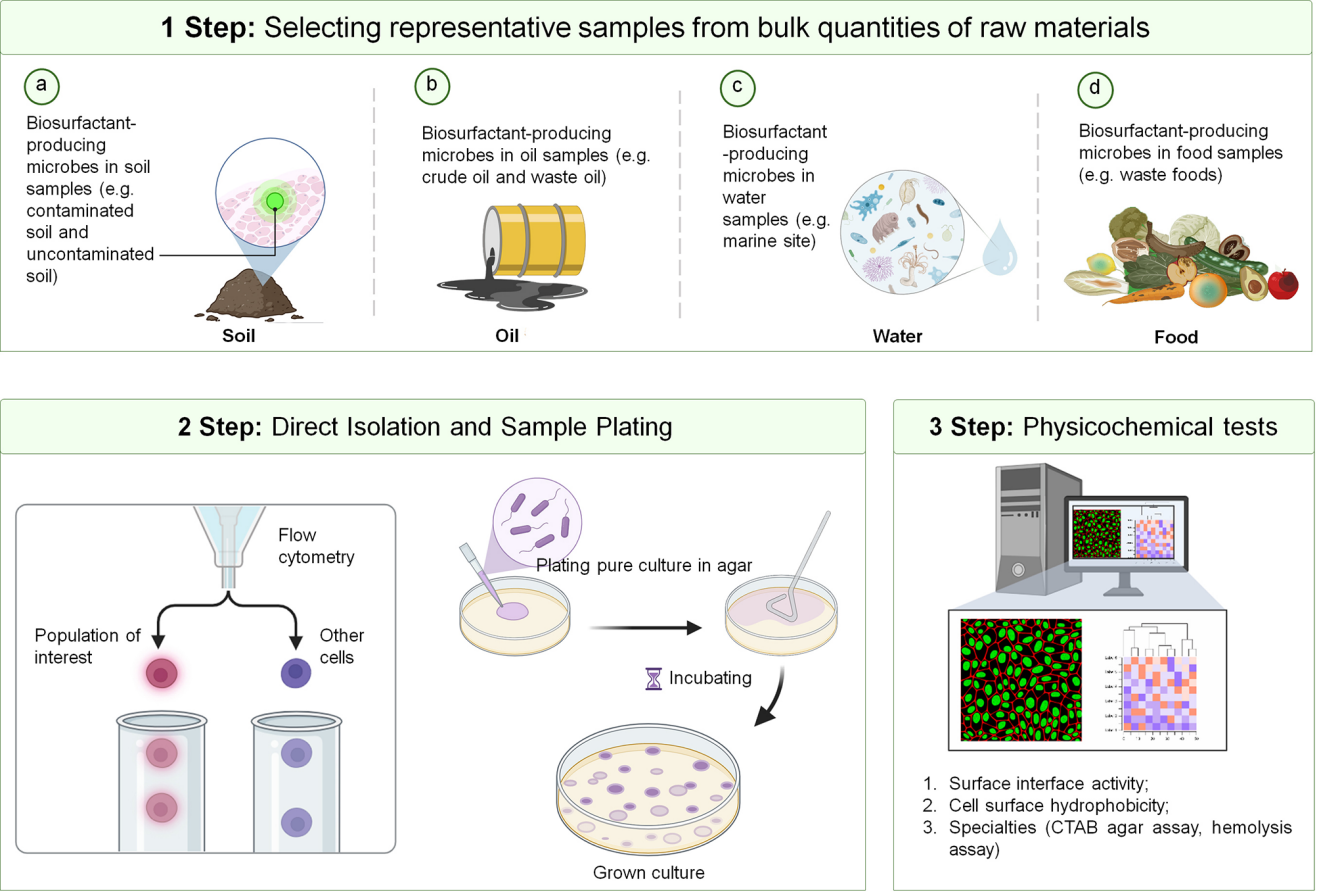
Complete strategy for screening new biosurfactant production

Currently, the market for commercially accessible biosurfactants is quite restricted, featuring only a few selections, including surfactin, sophorolipids, and rhamnolipids [41]. Consequently, there is a pressing need to intensify the search for novel biosurfactant-producing microorganisms. They can be found in soil [42–45], oil [46–51], water [52–55], and food [56–59]. The synthesis of biosurfactants is influenced by various factors, including water-soluble/insoluble carbon sources, nitrogen sources, pH, temperature, carbon-to-nitrogen (C/N) ratio, agitation, and oxygen availability [60–63]. The primary objectives of the screening process include identifying novel structures characterized by favorable physicochemical properties and detecting high-yield production strains [64, 65]. A complete strategy for screening new biosurfactant production can be seen in Fig. 4 [7].

A recent review focusing on the utilization of biosurfactants in oral hygiene applications noted that most of the examined biosurfactants for oral-related purposes belong to the lipopeptides or lipoproteins category. This investigation has revealed that the use of biosurfactants in oral health is in its nascent stage. Nevertheless, the published research in this field is promising and shows ongoing development [41].

The oral cavity continuously hosts oral microflora, which play a vital role in maintaining oral health. Disruptions in this equilibrium, whether caused by host factors or external influences, can create binding sites that opportunistic oral pathogens exploit, allowing them to dominate the oral cavity [66, 67]. Biosurfactants also play a role in quorum sensing and serve as antimicrobial agents involved in microbial competition [68, 69]. It is also crucial to uphold oral hygiene by consistently employing oral care products, such as toothpaste and mouthwash. These habits can effectively manage plaque development and suppress the proliferation of bacteria linked to dental diseases [70, 71].

One of the properties that need to be included in the toothpaste formula is good foaming ability since it allows the dentifrice to distribute evenly throughout the mouth during brushing and make thorough contact with tooth surfaces [72, 73]. This is typically accomplished by using a surface-active agent [74]. Incorporating biosurfactants into a toothpaste formulation can substantially diminish the need for chemical surfactants. Formulas containing biosurfactants demonstrated the ability to generate foam, suggesting that biosurfactants serve effectively as detergents in toothpaste [75, 76]. This finding aligns with the work of Das et al., who substituted SLS with biosurfactants from *Nocardiopsis VITSISB* in toothpaste [77]. Biosurfactants sourced from *Bacillus subtilis SPB1 (HQ392822)* in toothpaste formulations also reported can exhibit favorable characteristics, including strong foaming capabilities, effective stain removal properties on eggshells, an alkaline pH conducive to neutralizing acidic biofilms and demonstrates potent antimicrobial activity against the tested microorganisms [32]. Biosurfactants derived from *Pseudomonas aeruginosa UCP 0992* (PB) and *Candida bombicola URM 3718* (CB) combined with chitosan also exhibited significantly lower toxicity compared to commercial mouthwash products. These findings underscore the safety and effectiveness of natural product-based mouthwashes as a viable alternative for controlling oral microorganisms, providing a healthier option than commercially available mouthwashes [36].

Biofilm formation is related to all microbiological and chronic illnesses, particularly in oral and dental diseases, and is used by microorganisms to shield themselves from a hazardous environment [78, 79]. In normal physiological conditions, dental biofilm development involves the formation of a protein-rich acquired pellicle on dental surfaces, followed by the coaggregation and co-adhesion of various initial colonizers, such as *Streptococci* and members of the *Actinomyces* family [80–82]. Bridging colonizers such as *Fusobacterium* also contributes to this process by facilitating co-adhesion and coaggregation [83]. Typically, these biofilms consist predominantly of Gram-positive facultative anaerobes. However, inadequate hygiene can lead to an elevated percentage of Gram-negative species (e.g., *Porphyromonas spp*., *T. forsythia*, *Treponema denticola*, and *A. actinomycetemcomitans*) within the biofilms, thereby contributing to periodontal inflammation [84].

Recent advances in biofilm physiology have allowed researchers to learn more about bacterial biofilm inhibition [85]. There are two main inhibitory techniques, which are centered on the development of new antibiofilm chemicals and the development of biofilm-resistant surfaces [86]. Biosurfactants are the most promising choices for bacterial biofilm inhibition [5, 87, 88]. In heterogeneous systems, biosurfactants tend to aggregate at phase boundaries or interfaces, similar to how organic molecules in the aqueous phase immobilize at solid interfaces [89]. This aggregation forms a conditioning film, altering the surface properties such as surface energy and wettability and influencing the adhesion properties of microorganisms [90].

**Fig. 5:**
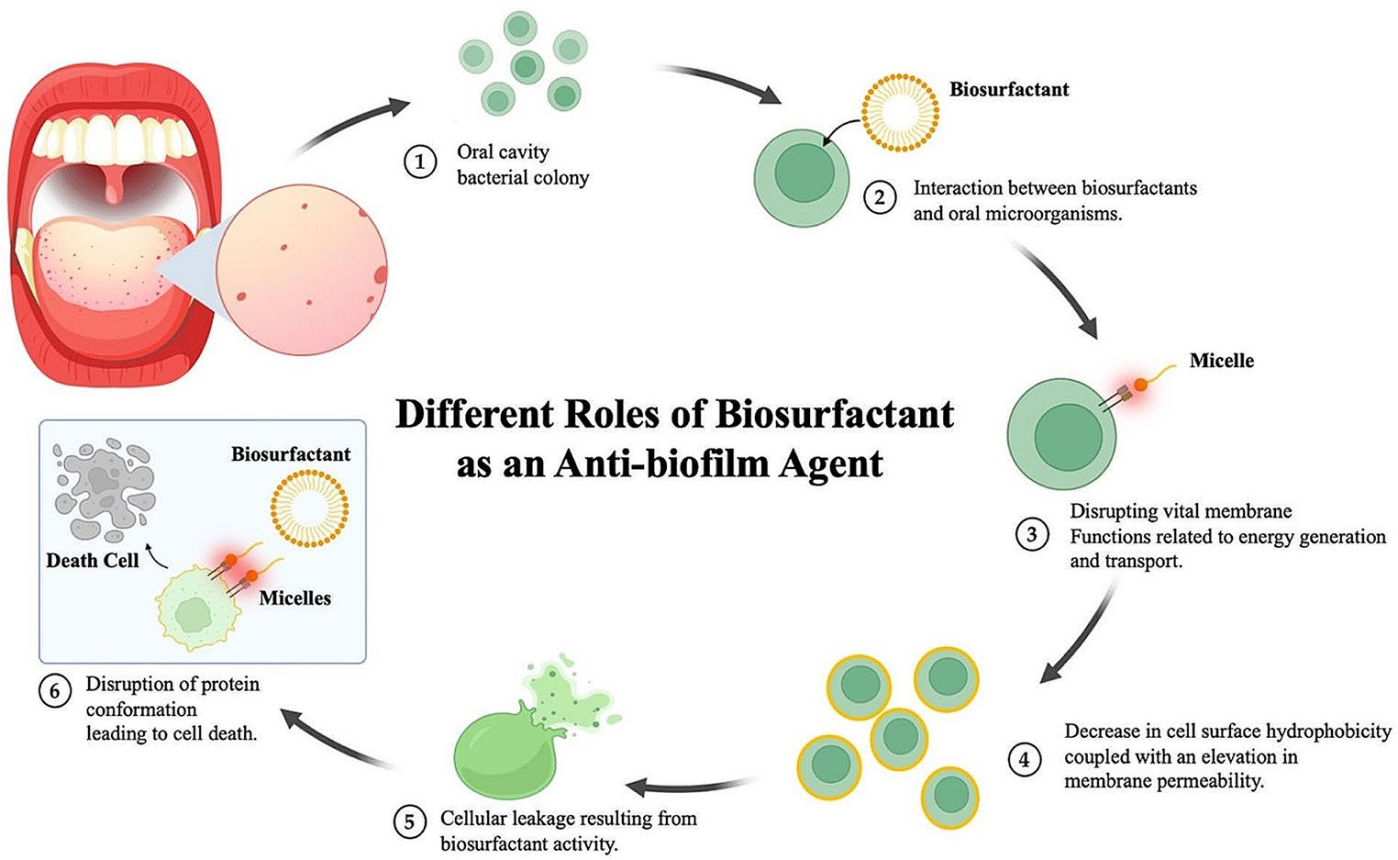
Different roles of biosurfactant as an anti-biofilm agent

Moreover, they can disrupt membranes, causing cell lysis by increasing membrane permeability, which leads to the leakage of cellular metabolites. This disruption can occur through changes in the physical membrane structure or by altering protein conformations that affect critical membrane functions like transport and energy generation. The role of biosurfactant as an anti-biofilm agent can be seen in Fig. 5 [91].

Rhamnolipids derived from the non-pathogenic *Burkholderia thailandensis E264* strain (ATCC 700,388) exhibit notable antibiofilm properties when tested in co-incubation experiments, pre-coated surface applications, and the disruption of immature biofilm against oral bacteria biofilms [27]. In vitro studies demonstrated that rhamnolipids can prevent and disrupt oral pathogen biofilms by increasing the permeability of oral pathogens in planktonic and oral biofilm states [35]. Rhamnolipids are reported to have the potential to inhibit the growth of oral bacteria and the formation of biofilms by *A. actinomycetemcomitans* Y4, making them a promising candidate for a novel oral drug to combat localized invasive periodontitis [40]. Surfactin was also reported to promote the antimicrobial activity of terpinen-4-ol (TP) against *S. mutans*, the causal agent of tooth decay, and can inhibit microbial pathogens’ growth and adhesion when combined with TP [33]. Lipopeptide biosurfactant demonstrates potent antimicrobial and anti-biofilm properties against *Enterococcus faecalis* grown in dentin specimens. It shows promise both as a standalone root canal irrigation solution and as an adjunct prior to the use of NaOCl in root canal treatments [92].

On implant applications, R89BS (biosurfactant extracted from *P. aeruginosa* 89) coating demonstrated effectiveness in reducing mixed biofilms of C. albicans and S. aureus on titanium surfaces, making it a promising approach for preventing microbial colonization on dental implants [38]. The same coating was applied to three different commercial implant surfaces, and the identical coating yielded a remarkable biomass inhibition exceeding 90% for *S. aureus* and reaching as high as 78% for *S. epidermidis* within 24 h [39].

In terms of the dose, some papers reported biosurfactants showed dose-dependent characteristics. Biosurfactants obtained from *Lactobacillus acidophilus DDS-1*, *Lactobacillus rhamnosus ATCC 53,103*, and *Lactobacillus paracasei B21060* exhibited substantial inhibition of adhesion and biofilm formation on titanium surfaces by *S. mutans* and *S. oralis* in a dose-dependent manner. This was evident from the significant reduction in cfu/ml values and biomass production [34]. Elshikh M. et al. also reported that higher rhamnolipid concentrations can increase the permeabilization effects on both the gram-positive and gram-negative bacteria used in their study [27]. Both Surfactin C_-15_ (SF) and metal(II)-SF complexes demonstrated a concentration-dependent inhibition of biofilm formation and a reduction in the metabolic activity of mature biofilms that led to a decrease in the mRNA expression of hypha-specific genes (e.g., HWP1, ALS1, ALS3, ECE1, and SAP4) without causing significant growth inhibition of *C. albicans* [37]. Lipopeptide biosurfactant (F7) extracted from *Bacillus clausii* also demonstrated dose-dependent against *S. mutans*, *E. faecalis*, and *C. albicans*. Higher F7 biosurfactant concentrations showed greater inhibition percentages [93].

The limitation of this systematic review is that numerous studies do not provide sufficient evidence regarding the thorough purity or comprehensive characterization of the active biosurfactant fractions they employ. This issue is exemplified by the research conducted by Tahmourespour et al. (2011) [94], Tahmourespour, Salehi, and KasraKermanshahi (2011) [95], and Salehi et al. (2014) [96], Savabi et al. (2014) [97], during their investigation of the gene expression of gtfB, gtfC, and ftf in *S. mutans* which directly involved in the formation of biofilm matrices. Notably, these studies are pioneering efforts as they represent the first instances of exploring the gene expression of oral-related bacteria following treatment with biosurfactants. Other limitations include the relatively short timeframe covered by this systematic review. The choice of this timeframe was motivated by the need to present the most current research papers exploring biosurfactants’ use in oral applications. Interpreting the results of in vitro studies presents challenges due to variations in the methods used for material preparation and microbial exposure across different studies. This is crucial because data comparison becomes arduous without standardized methods, and drawing meaningful conclusions and extrapolating findings becomes problematic. Deviating from these recommendations in experiments may limit the applicability of the results.

## Conclusion

This systematic review suggests that biosurfactants hold significant promise for oral applications. Their properties, such as antimicrobial and antibiofilm activity against both gram-positive bacteria, gram-negative bacteria, and fungi, the ability to form stable or metastable microemulsions, and their capacity to enhance the bioavailability of hydrophobic compounds, make biosurfactants attractive candidates for use in cosmetic or therapeutic oral hygiene products, as well as oral-related medical devices. Utilizing biosurfactants alone or combined with other antimicrobial or chemotherapeutic agents presents a promising strategy for preventing and combating microbial infections, biofilm formation, and proliferation.

### Perspectives and future directions

Biosurfactants have recently gained attention within the scientific community as a promising oral application addition to the next generation. However, to fully realize the potential biosurfactants, substantial efforts are required to improve the quality of research in this area. Enhancing research quality may help attract skeptical industrial collaborators. When attributing bioactivity to biosurfactants, it is crucial to use high-purity biosurfactants. It is also crucial to emphasize that their multifaceted properties can interact and potentially lead to side effects in various applications, necessitating thorough investigation. At the same time, the commercial utilization of biosurfactants is becoming increasingly pertinent and essential to mitigate the environmental impact associated with conventional synthetic surfactants. Nevertheless, challenges related to cost-effectiveness and availability of biosurfactants for potential applications still require resolution.

## Data Availability

The data supporting the findings of this study are available upon request from the corresponding author, MNZ.
